# Posttransplant lymphoproliferative disorders following hematopoietic stem cell transplantation: a clinicopathological study of a large single-center cohort

**DOI:** 10.1007/s00277-026-07225-4

**Published:** 2026-08-05

**Authors:** Jun Lin, Mengyun Zhou, Chang Shen, Feng Peng, Cheng Xu, Qian Huang

**Affiliations:** 1 Pathology Center, Shanghai Genenal Hospital, Shanghai Jiaotong University School of Medicine, Shanghai, 200080 China; 2 Department of Pathology, Bengbu Hospital of Shanghai Genenal Hospital/The Second Affiliated Hospital of Bengbu Medical University, Bengbu, Anhui 233000 China; 3 Department of Pathology, Shanghai Eighth People’s Hospital, Shanghai, 200235 China; 4 Department of Hematology, Shanghai General Hospital, Shanghai Jiaotong University School of Medicine, Shanghai, 200080 China; 5 Cancer Center, Shanghai General Hospital, Shanghai Jiaotong University School of Medicine, No.100 Hai Ning Road, Hong’kou District, Shanghai, 200080 China

**Keywords:** PTLD, HSCT, EBV, Histomorphology, Immunohistochemistry, Rituximab

## Abstract

Posttransplant lymphoproliferative disorder (PTLD) is a serious complication of hematopoietic stem cell transplantation (HSCT) and is typically driven by immunosuppression and Epstein–Barr virus infection. This study analyzed the clinicopathological spectrum and outcomes of PTLD in a large single-center cohort. We retrospectively reviewed 37 PTLD patients who were diagnosed post-HSCT (2017–2024). Histopathological classification was conducted in accordance with the 5th Edition of the World Health Organization Classification of Hematolymphoid Tumors (WHO-HAEM5). Immunohistochemistry, EBV-encoded RNA in situ hybridization, fluorescence in situ hybridization, and clonality assays were performed. Clinical data, treatment responses, and survival outcomes were collected and analyzed. The PTLD incidence was 2.4% among allo-HSCT recipients, with a male predominance (2.36:1). The median time to onset post-HSCT was 3 months (range: 1–21 months), with 81.1% of cases occurring within 6 months. The pathological types of PTLD included hyperplasias (9 cases), polymorphic PTLD (20 cases), and lymphoma (8 cases; all diffuse large B-cell lymphomas). First-line management include reduced immunosuppression, rituximab, and antiviral therapy. Prebiopsy rituximab in 5 patients caused CD20 loss, complicating the diagnosis. The mortality rate was 27%, primarily from infectious complications following PTLD-directed therapy. The median postdiagnosis survival was 3.5 months. PTLD post-HSCT is a serious complication that often follows an aggressive clinical course. While the direct contribution of PTLD to mortality was challenging to establish, its development frequently triggered intensive interventions (e.g., rituximab and immunosuppression reduction), which in turn led to fatal infectious complications in more than one-quarter of our cohort. Therefore, the high mortality in these patients was largely indirect and attributable to treatment-related infectious complications in profoundly immunocompromised hosts.

## Introduction

Posttransplant lymphoproliferative disorders (PTLDs) are a heterogeneous group of lymphoid or plasmacytic proliferations that develop because of chronic immunosuppression in solid organ transplant (SOT) and hematopoietic stem cell transplant (HSCT) recipients [1]. Although it is relatively rare, PTLD is associated with significant morbidity and mortality and poses considerable challenges to clinical management. Its incidence varies widely, from 1% to 20% in SOT recipients [2] and is < 2% in HSCT recipients [3]. With the increasing number of organ transplantation operations and the widespread use of immunosuppressants after transplantation, the incidence of PTLD has shown an increasing trend in recent years.

The diagnosis and classification of PTLD have evolved significantly. The 5th Edition of the World Health Organization Classification of Hematolymphoid Tumors (WHO-HAEM5) introduces a new standardized nomenclature to address inconsistencies in terminology and diagnostic criteria for similar lesions across different immunodeficiency settings [4]. On the basis of histological diagnosis, PTLDs were previously classified into four major categories: nondestructive PTLD, polymorphic PTLD (P-PTLD), monomorphic PTLD, and classical Hodgkin lymphoma-type PTLD. However, the new classification system consolidates these into three main histological types: hyperplasias (HP), P-PTLD and lymphomas.

However, diagnosing PTLD remains challenging because of its diverse clinical manifestations, morphological heterogeneity, and overlap with other types of lymphoid proliferation. Additionally, the rapid progression of the disease often necessitates urgent intervention, sometimes even before a definitive pathological diagnosis is established. The role of Epstein–Barr virus (EBV) infection is well recognized, yet not all cases are associated with EBV, which further complicates the diagnostic process. Moreover, the increasing use of preemptive therapies, such as rituximab, can alter pathological features, potentially obscuring accurate histological assessment.

Given these complexities, there is a pressing need to improve the understanding of the clinicopathological characteristics of PTLD to facilitate early and accurate diagnosis. Capitalizing on the clinical strengths of our hospital’s hematology department, a top-tier center for HSCT procedures in Shanghai, particularly allogeneic HSCT (allo-HSCT), we retrospectively analyzed 37 cases of PTLD following HSCT over a 8-year period. This article aims to elucidate the clinical and pathological features, diagnostic approaches, underlying pathogenesis, and treatment strategies for PTLD, with the goal of improving diagnostic accuracy and informing clinical management.

## Materials and methods

### Patients

Patients who were diagnosed with PTLD after HSCT at the Pathology Center of the Shanghai General Hospital between January 2017 and December 2024 were included. The diagnosis was based on the WHO-HAEM5 criteria [4] and was confirmed by 2 experienced pathologists (JL and MZ). Follow-up data were collected from electronic medical records and telephone interviews. From January 2017 to December 2024, a total of 1,544 patients underwent allo-HSCT at our center, among whom 37 developed PTLD, yielding an overall incidence of 2.4% (37/1,544), or 24.0 cases per 1,000 transplants. This study was approved by the Shanghai General Hospital Institutional Review Board (approval no. 2025010) and was conducted in accordance with the Declaration of Helsinki. This study utilized archived biological samples and medical records and was exempt from the need for informed consent. All the experiments were conducted in accordance with the relevant guidelines and regulations.

### Histology and immunohistochemistry (IHC)

All tissue samples were fixed with 10% neutral formalin, desiccated, embedded in paraffin, and stained with hematoxylin and eosin (HE). Stained sections were used to assess histopathological features. The tissue blocks were cut into 4-µm sections and processed for immunohistochemistry. Immunohistochemical staining was performed by using an automated immunostainer (Ventana Medical Systems) following the manufacturer’s protocol. Appropriate positive and negative controls were used in all the experiments. Antibodies against CD20, CD79a, PAX5, CD19, CD3, CD5, CD2, CD7, CD138, CD38, MUM-1, CD10, CD23, BCL2, BCL6, CD43, CD30, CD4, CD8, TDT, kappa light chain, lambda light chain, and Ki67 were used.

### EBER-ISH, fluorescence in situ hybridization (FISH) and clonality analysis

EBV was detected with a BOND ready-to-use ISH EBER probe (Leica Biosystems Newcastle, Ltd.). Nasopharyngeal carcinoma tissue was used as the positive control on each slide.

FISH was performed on formalin-fixed, paraffin-embedded (FFPE) tissue sections from patients with a morphological diagnosis of DLBCL in whom the tumor cells exhibited medium-sized or blastoid morphology, raising the morphological suspicion of high-grade B-cell lymphoma with possible MYC, BCL2, and/or BCL6 rearrangements. FISH was selectively applied to these cases to exclude the possibility of high-grade B-cell lymphoma (including double-hit/triple-hit lymphoma), as is routine in our diagnostic workflow. The following dual-color probes were used: 3q27 (BCL6 break probe), t(14;18)(q32;q21) (IGH::BCL2 fusion probe), and 8q24 (MYC break probe) (all probes from LBP Medicine Science & Technology Co., Ltd., Beijing, China).

DNA was extracted from FFPE samples. Clonality analysis was performed via polymerase chain reaction (PCR) according to the BIOMED-2 PCR protocol (Shanghai Yuanqi Biomedical Technology Co., Ltd), as described elsewhere [5]. To assess B-cell clonality, multiplex PCR assays were used to target the immunoglobulin heavy chain (IGH) gene framework regions and the immunoglobulin kappa/lambda (IGK/IGL) light chain genes. To assess T-cell clonality, multiplex PCR assays were used to target the T-cell receptor gamma (TCRG) and beta (TCRB) chain genes.

### Statistical analysis

The clinicopathological, immunohistochemical, and FISH data were analyzed descriptively. Categorical variables are presented as absolute numbers and percentages. Continuous variables, such as the time from transplantation to PTLD onset, were compared via the Mann‒Whitney U test (for nonnormally distributed data). A p value of less than 0.05 was considered to indicate statistical significance. Statistical analyses were performed by using SPSS version 23.0.

## Results

### Clinical features

The baseline clinical characteristics and transplantation-related variables, including conditioning regimen intensity, total body irradiation (TBI) use, graft source, and GVHD prophylaxis regimens, are summarized in Table 1. There was a male predominance, with a male-to-female ratio of 2.36:1 (26 men and 11 women). The patient ages ranged from 16 to 70 years (mean: 38.37 years; median: 37 years; interquartile range: 28.5–46.5 years). All the patients had received allo-HSCT and had underlying disease, including acute myeloid leukemia (14 cases), acute lymphoblastic leukemia (7 cases), T-cell lymphoma (7 cases), aplastic anemia (6 cases), and myelodysplastic syndrome (3 cases). Thirty-three patients received HSCT from mismatched related donors (MMRDs), and four received HSCT from unrelated donors. One patient underwent allo-HSCT 8 months after autologous transplantation for T-cell lymphoma. The median time interval from transplantation to PTLD was 3 months (mean: 4.14 months; range: 1–21 months; interquartile range: 2.00–4.38 months). Notably, 81.1% of the patients (30/37) were diagnosed within 6 months, 70.3% (26/37) within 4 months, and 21.6% (8/37) within 2 months post-HSCT. All patients received standard antimicrobial prophylaxis according to our institutional protocol, including (1) antibacterial prophylaxis with levofloxacin; (2) antifungal prophylaxis with posaconazole; (3) antiviral prophylaxis with acyclovir/valacyclovir or ganciclovir; and (4) Pneumocystis jirovecii prophylaxis with trimethoprim-sulfamethoxazole.

### Histological and immunophenotypic features

Forty-five biopsy samples, primarily from lymph nodes and mucosal tissue, were obtained from the 37 patients. Thirty-eight specimens were from the lymph nodes and included 12 surgical excisional biopsy (SEB) specimens and 26 core needle biopsy (CNB) specimens. The remaining specimens included 5 from the nasopharynx, 1 from the tonsil, and 1 from the lung. Eight patients contributed two specimens each.

Microscopically, our cases could be divided into three morphological categories: HP, P-PTLD and lymphoma.

**Fig. 1:**
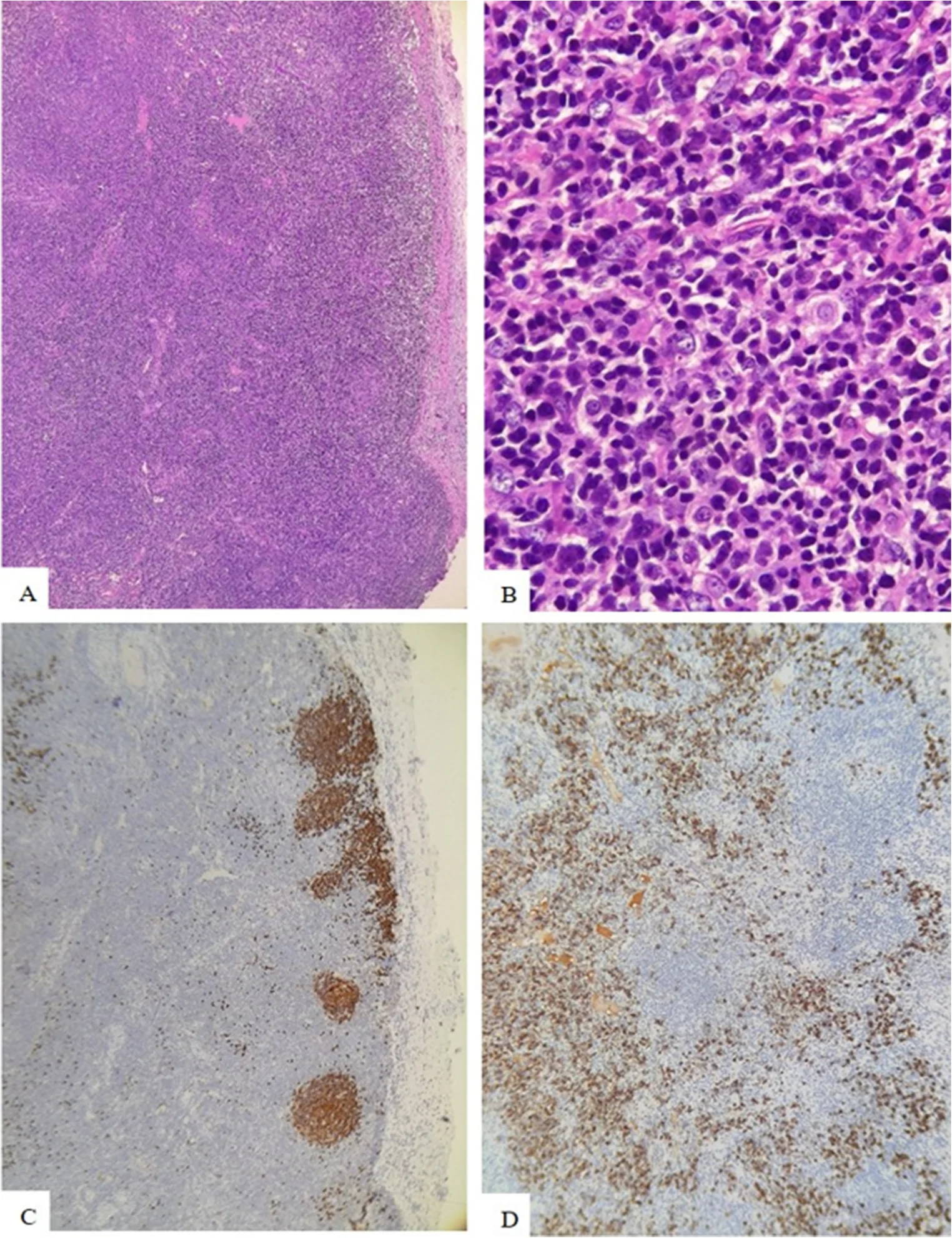
Plasmacytic hyperplasia (PCH). **A** Low-power field examination revealed preserved lymph node architecture. The follicles were atrophic with expanded interfollicular areas (HE, 100×). **B** In a high-power field, the proliferation of plasma cells and plasmacytoid cells was detected (HE, 400×). **C** The atrophic follicles were positive for CD20 (IHC, 100×). **D** The number of CD138-positive plasma cells was increased in the interfollicular areas (IHC, 200×)

**Fig. 2:**
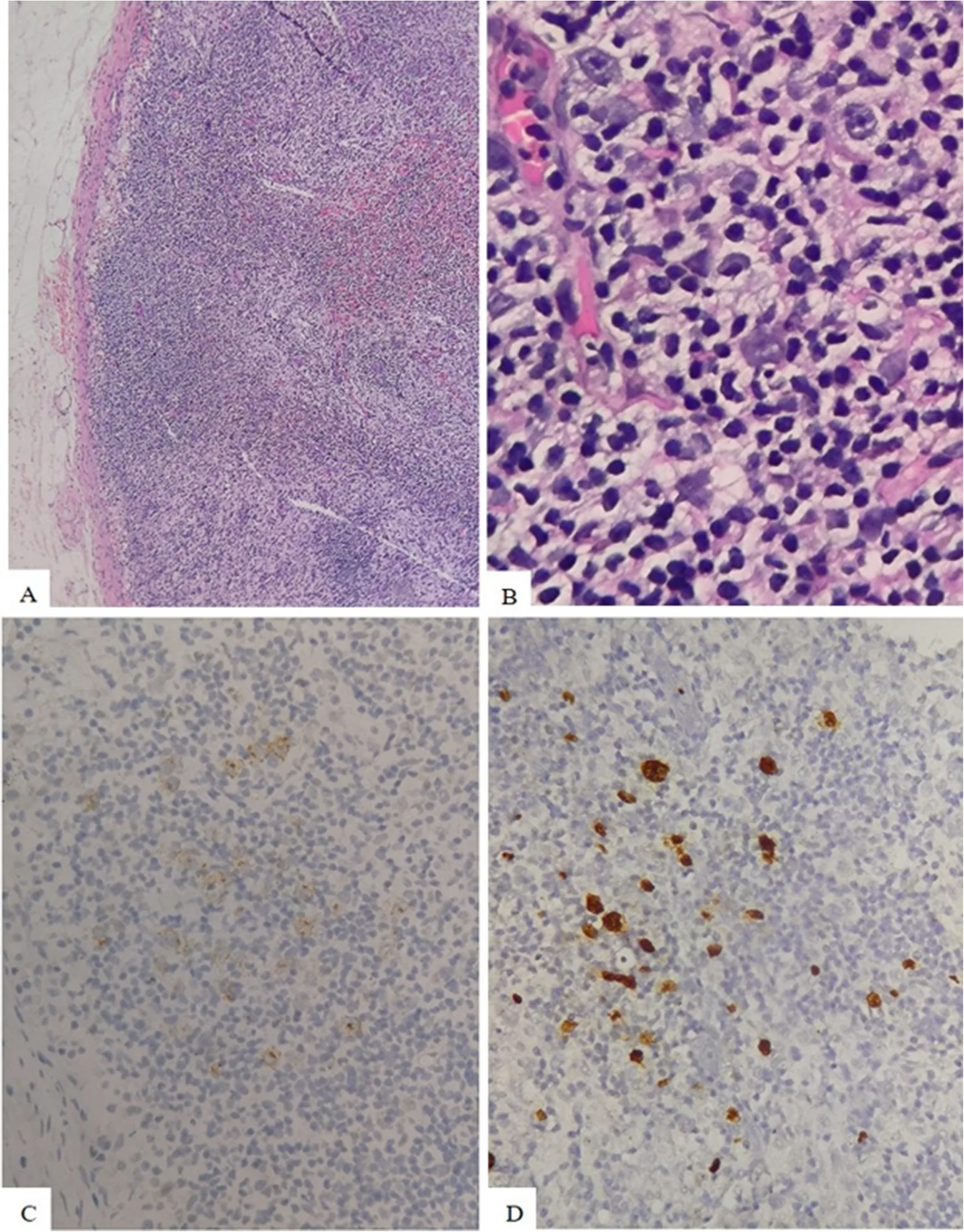
Infectious mononucleosis-like hyperplasia (IMH). **A** Paracortical expansion (HE, 200×). **B** Increased immunoblasts on a background of small lymphoid cells, plasma cells, and plasmacytoid cells (HE, 400×). **C** Immunoblasts were weakly positive for CD30 (IHC, 200×). **D** In situ hybridization revealed scattered EBER-positive cells (ISH, 200×)

Nine cases in which the architecture of the affected lymphoid organ was preserved were classified as HP. Among them, 7 cases of plasmacytic hyperplasia (PCH) showed infiltration of plasma cells and plasmacytoid cells in the interfollicular area (Fig. 1). Two cases of infectious mononucleosis-like hyperplasia (IMH) exhibited paracortical expansion with increased immunoblasts on a background of small lymphoid cells, plasma cells, histiocytes and eosinophils (Fig. 2).

**Fig. 3:**
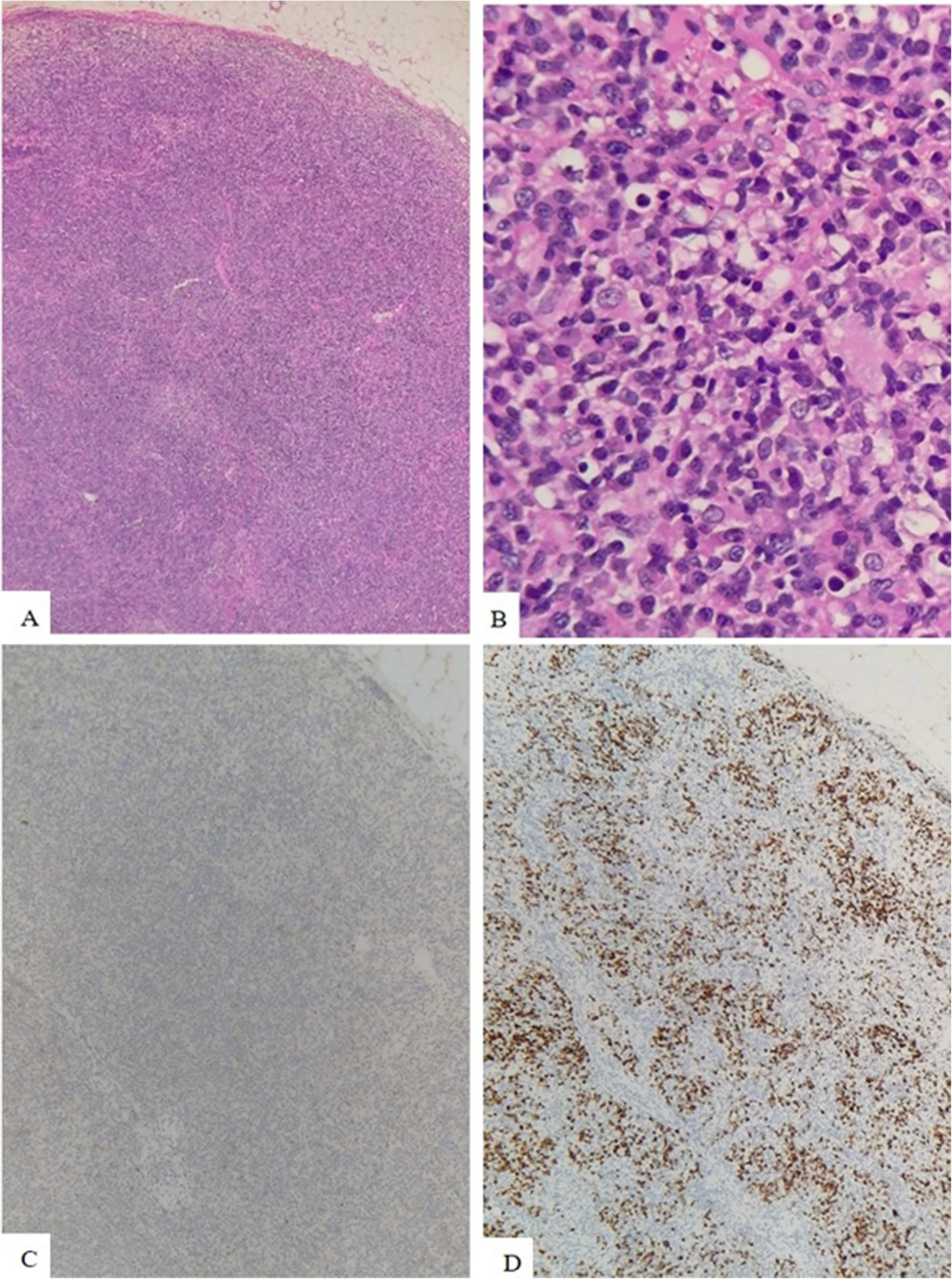
P-PTLD. **A** The nodal architecture was effaced (HE, 100×). **B** Polymorphic B cells infiltrated with immunoblasts, plasmacytoid cells, and plasma cells, along with reactive T cells (IHC, 400×). **C** The biopsy sample obtained four days after rituximab administration showed a loss of CD20 expression (IHC, 100×). **D** CD79a immunohistochemistry highlights B lymphocytes, plasma cells, and plasmacytoid cells (IHC, 200×). The patient died of the disease 3 months after the diagnostic biopsy

**Fig. 4:**
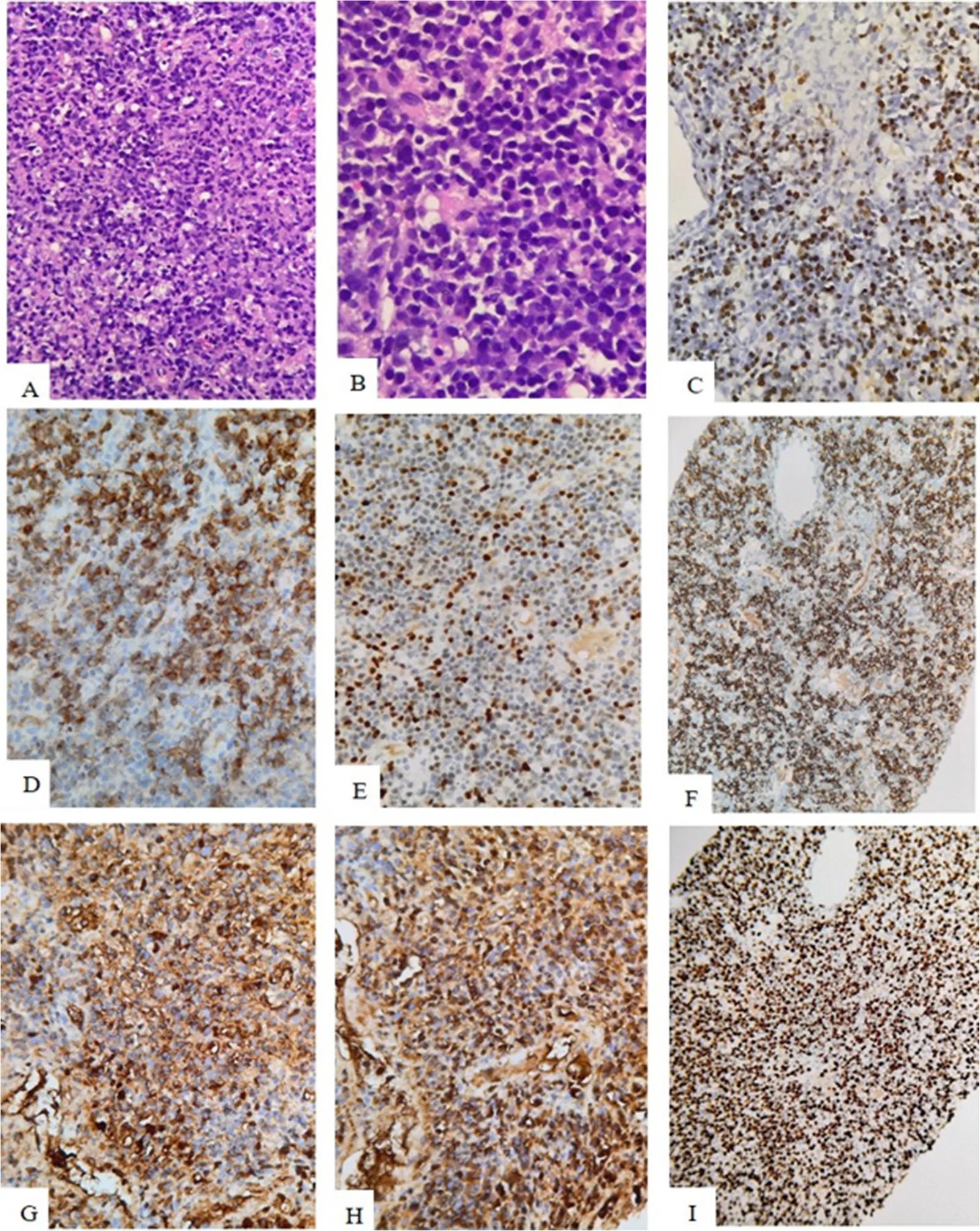
P-PTLD. **A** Effacement of the normal lymphoid architecture in a core needle biopsy sample (HE, 200×). **B** High-power examination revealed predominantly small- to medium-sized cells with scattered large cells (HE, 400×). **C** EBER-positive cells (~ 50%) (ISH, 200×). **D** CD20 (IHC, 200×) and **E** PAX5 (IHC, 200×) staining. **F** CD138 immunohistochemistry highlights prominent plasmacytic and plasmacytoid cell proliferation (IHC, 200×). Unrestricted expression of **G** kappa (IHC, 200×) and **H** lambda (IHC, 200×) light chains. **I** The Ki67 proliferation index was determined for approximately 80% of the cells (IHC, 200×)

Twenty cases were classified as P-PTLD, a subtype of lymphoproliferative disorder of varied malignant potential, which is characterized by a heterogeneous cellular infiltrate that affects the normal tissue architecture (Fig. 3). Geographic necrosis was present in 5 patients. The lesions contained variable proportions of B cells (CD20+) at different stages of differentiation. Immunostaining revealed a heterogeneous lymphoid population, including polyclonal B cells (CD20+), plasmacytoid cells (CD138 + and/or CD38 + and MUM-1+), immunoblasts (CD30+), and mature plasma cells (CD138 + CD38+) (Fig. 4), along with scattered reactive T cells (CD3+) and histiocytes (CD68+).

**Fig. 5:**
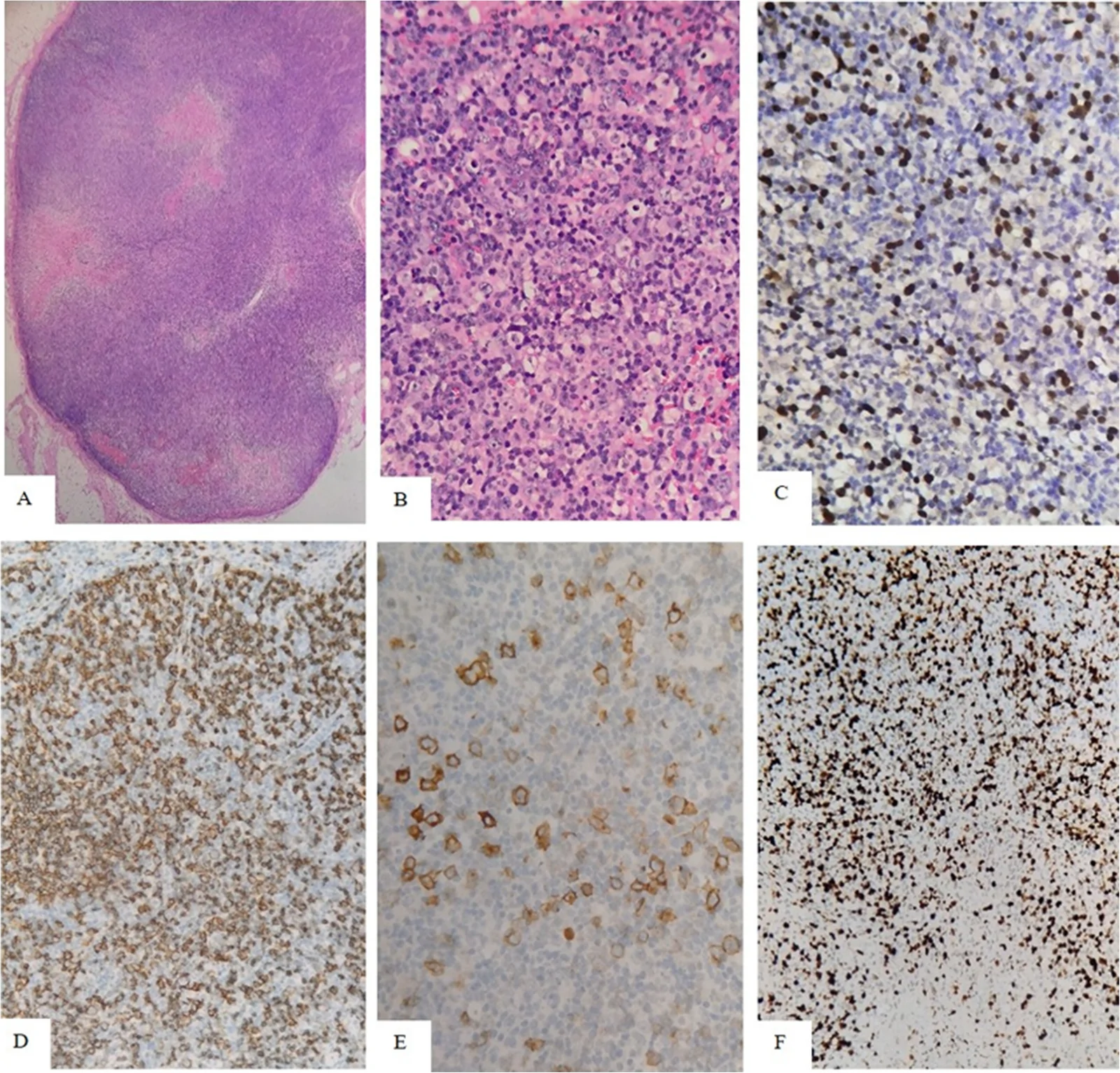
DLBCL. **A** The nodal architecture was effaced with geographic necrosis (HE, 100×). **B** Sheets of large atypical cells are present. Scattered small lymphocytes, plasmacytoid cells, and plasma cells, along with reactive T cells (IHC, 400×). C EBER-positive cells (~ 30%) (ISH, 200×). **D** CD20 expression was diffuse in the tumor cells (IHC, 200×). **E** Large atypical cells were positive for CD30 (IHC, 400×). **F** The Ki67 proliferation index was determined for approximately 60% of the cells (IHC, 200×)

Eight patients were diagnosed with DLBCL. The key diagnostic feature was the presence of diffuse sheets of large B cells. These cases contained large transformed cells and immunoblasts, reactive components, and Hodgkin/Reed–Sternberg (HRS)-like cells (CD20 + CD30+). An increased number of plasma cells (CD138+) was sometimes observed. Geographic necrosis was also noted in two patients (Fig. 5).

The clinicopathological characteristics of the 37 PTLD cases, including histological subtypes, time from transplantation to onset, EBV/CMV status, GVHD, and survival outcomes, are summarized in Table 2.

One patient submitted two specimens from different lymph node sites. The initial CNB was diagnosed as P-PTLD, while the subsequent SEB was diagnosed as DLBCL. For analysis, this patient was categorized as having DLBCL. Analysis of the SEB specimen revealed clonal rearrangements of IGH and IGK.

Another patient who had two specimens underwent one before and one after rituximab treatment, and both were diagnosed with DLBCL. The posttreatment sample (obtained two months after rituximab treatment) was CD20 negative but remained positive for other B-cell markers (PAX5 + CD79a+). Additionally, EBER-ISH was negative. The other six patients who submitted two specimens each had consistent diagnoses between the two samples.

Biopsy samples were taken from 5 patients who had received rituximab treatment 1 to 5 days prior. CD20 immunostaining was diminished or lost in all the samples (Fig. 3). Among these patients, three were diagnosed with P-PTLD, one with DLBCL and one with IM.

### Results of EBER-ISH, FISH and clonality analysis

EBER-ISH was positive in 34 of the 37 patients (91.9%) (Figs. 2, 4, and 5). The time from transplantation to PTLD onset in the EBER-negative patients (median: 6.5 months; range: 1.5–21 months) appeared to be longer than that in the EBER-positive patients (median: 3 months; range: 1–7.25 months). However, this difference was not statistically significant (*p* = 0.15; Mann‒Whitney U test), likely because of the very small number of EBER-negative patients (*n* = 3). The clinicopathological features of these three EBER-negative patients are summarized in Table 3. On the basis of their histological features, posttransplant clinical context, and molecular findings, all three cases were considered EBV-negative PTLD rather than de novo lymphomas unrelated to transplantation. Specifically, Case 1 was classified as DLBCL (a monomorphic PTLD subtype), while Case 2 was classified as PCH, and Case 3 was classified as P-PTLD, with neither case exhibiting the diagnostic features of lymphoma.

FISH analysis was performed for three patients, all of whom had a morphological diagnosis of DLBCL. No rearrangements of MYC, BCL2 or BCL6 were detected.

Clonality analysis of IGH, IGK and T-cell receptor gene rearrangements was performed for 5 patients. Clonal IGH and IGK rearrangements were detected in 3 patients (2 with DLBCL and 1 with P-PTLD). Clonal IGH and IGK rearrangements and suspected TCR positivity were found in 2 patients with P-PTLD.

**Fig. 6:**
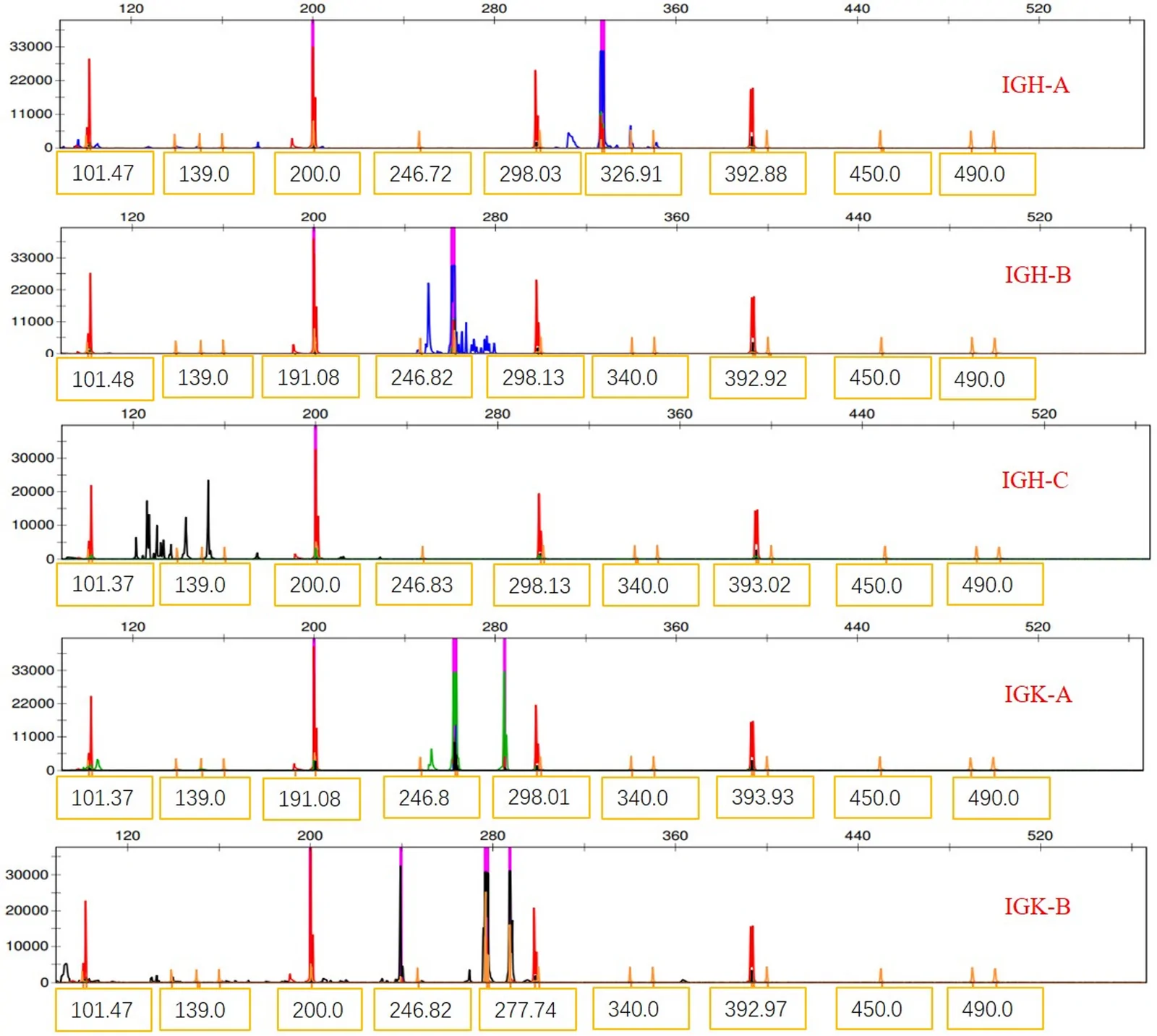
P-PTLD. Monoclonal rearrangements were detected in both the IGH and IGK genes in a patient with a diagnosis of P-PTLD (the same patient as in Fig. 4). The blue fluorescence indicates the monoclonal proliferation products of IGH-A (295–378 bp) and IGH-B (236–307 bp). The green fluorescence indicates the monoclonal proliferation products of IGK-A (120–160, 190–210, and 260–300 bp). The black fluorescence indicates the monoclonal proliferation products of IGK-B (210–250, 270–300, and 350–390 bp)

Seven patients who were tested only for IGH and IGK rearrangements, including 4 with P-PTLD (Fig. 6) and 3 with DLBCL, were positive.

Two patients with a history of primary T-cell lymphoma before transplantation underwent only TCR gene rearrangement testing, which yielded negative results.

### Treatment and follow-up

With a median follow-up of 15 months (range: 1–96 months) for the entire cohort, 10 of the 37 patients (27.0%) died. The median overall survival (OS) was not reached. The mean time from diagnosis to death among the 10 deceased patients was 3.5 months (range: 1–7 months). Notably, the causes of death were primarily treatment-related complications; eight patients died from severe infections (e.g., pulmonary infection and respiratory failure), and two died from the relapse of their primary underlying malignancy. Among the eight patients who died of infectious complications, seven were EBV positive, three had cytomegalovirus (CMV) infection, and one had acute GVHD. Seven of these eight patients received antiviral therapy combined with immunosuppression reduction, while one patient with acute GVHD received antiviral therapy with intensified immunosuppression. All eight patients were treated with rituximab. No deaths were directly attributed to uncontrolled PTLD progression. Three patients were lost to follow-up after 14, 21 and 8 months. EBV and CMV loads were monitored during treatment. Thirty patients had elevated EBV loads, and five had CMV infection. Graft-versus-host disease (GVHD) occurred in 4 patients, including 3 with acute GVHD and 1 with chronic GVHD. Immunosuppressants were reduced in 28 patients, and antiviral therapy (AVT) was used in 33 patients; both interventions were typically initiated before biopsy. The anti-CD20 monoclonal antibody rituximab was used in 30 patients, with the first dose administered before biopsy in 5 patients (1–5 days). Treatment strategies and outcomes stratified by histological subtype are summarized in Table 4. Among the three groups, the combination of immunosuppression reduction, antiviral therapy, and rituximab (ISR + AVT + R) was the most commonly used regimen across all subtypes, particularly in DLBCL patients (7/8, 87.5%). Patients with HP had the most favorable outcomes, with only 1 death (11.1%) during follow-up. In contrast, P-PTLD patients had a mortality rate of 35.0% (7/20), and DLBCL patients had a mortality rate of 25.0% (2/8). EBER positivity rates were consistently high across all subtypes (88.9%–95.0%), which is consistent with the strong association between EBV and PTLD in the post-HSCT setting. These findings suggest that while most patients received similar treatment regimens, the outcomes varied by histological subtype, with hyperplasias having the best prognosis and P-PTLD having the worst prognosis.

**Table 1:** Baseline characteristics and transplantation related variables

Feature	Patients	%
All patients	37	-
Mean age, years (range)	38.4(16–70)	-
Sex		
Male	26	70.3
Female	11	29.7
Primary disease		
AML	14	37.8
ALL	7	18.9
TCL	7	18.9
AA	6	16.2
MDS	3	8.1
Donor type		
Mismatched Related donor	33	89.2
Unrelated donor	4	10.8
Graft source		
PBSC	32	86.5
CBSC+PBSC	5	13.5
Conditioning regimen intensity		
MAC	29	78.4
RIC	8	21.6
TBI use		
With TBI	16	43.2
Without TBI	21	56.8
GVHD prophylaxis regimen		
ATG + CsA + PTCy + MMF/MPA	22	59.5
ATG + CsA + MTX + MMF/MPA	10	27.0
ATG + CsA + MTX	3	8.1
ATG + Tac + MTX + MMF	1	2.7
ATG + CsA +MMF	1	2.7

*AML,* acute myeloid leukemia; *ALL,* acute lymphoblastic leukemia; *TCL,* T-cell lymphoma; *AA,* aplastic anemia; *MDS,* myelodysplastic syndrome; *PBSC,* peripheral blood stem cell; *CBSC,* cord blood stem cell; *MAC,* myeloablative conditioning; *RIC,* reduced-intensity conditioning; *TBI,* total body irradiation; *GVHD,* graft-versus-host disease; *ATG,* anti-thymocyte globulin; *CsA,* cyclosporine A; *PTCy,* posttransplant cyclophosphamide; *MMF,* mycophenolate mofetil; *MPA,* mycophenolic acid; *MTX,* methotrexate; *Tac,* tacrolimus

**Table 2:** Clinicopathological characteristics of the PTLD patients

Feature	Patients	%
Histological type		
IMH	2	5.4
PCH	7	18.9
P-PTLD	20	54.0
DLBCL	8	21.6
Time from transplantation to PTLD		
≤ 2 months	8	21.6
2.1–4 months	18	48.6
4.1–6 months	4	10.8
> 6 months	7	18.9
Blood EBV DNA		
+	30	81.1
-	7	18.9
CMV infection		
+	5	13.5
-	32	86.5
GVHD		
Acute	3	8.1
Chronic	1	2.7
-	33	89.2
Survival status		
Live	24	64.9
Dead	10	27.0
Unknown	3	8.1

*AML,* acute myeloid leukemia; *ALL,* acute lymphoblastic leukemia; *TCL,* T-cell lymphoma; *AA,* aplastic anemia; *MDS,* myelodysplastic syndrome; *IMH,* infectious mononucleosis-like hyperplasia; *PCH,* plasmacytic hyperplasia; *P-PTLD,* polymorphic posttransplant lymphoproliferative disorder; *DLBCL,* diffuse large B-cell lymphoma; *PCN,* plasma cell neoplasm; *EBV,* Epstein‒Barr virus; *CMV,* cytomegalovirus; *GVHD,* graft-versus-host disease

**Table 3:** Clinicopathological features of EBER-negative cases

Feature	Case 1	Case 2	Case 3
Primary disease	AA	TCL	ALL
Histological type	DLBCL	PCH	P-PTLD
Time to PTLD (months)	1.5	21	6.5
EBER-ISH	-	-	-
Blood EBV DNA	+	-	-
CMV infection	+	-	-
IGH/IGK Clonality	+	ND	-
TCR Clonality	ND	-	ND
Treatment	AVT, ISR, R	Untreat	R
Survival status	Dead	Live	Live
Outcome (months)	2	55	11

*AA,* aplastic anemia; *TCL,* T-cell lymphoma; *ALL,* acute lymphoblastic leukemia; *DLBCL,* diffuse large B-cell lymphoma; *PCH,* plasmacytic hyperplasia; *P-PTLD,* polymorphic posttransplant lymphoproliferative disorder; *AVT,* antiviral therapy; *ISR,* immunosuppression reduction; *R,* rituximab; *ND,* Not Done

**Table 4:** Treatment strategies and outcomes stratified by histological subtype

Feature	HP (9)	*P*-PTLD (20)	DLBCL (8)	Total (37)
Treatment strategy				
ISR	0	0	1 (12.5%)	1 (2.7%)
AVT	2 (22.2%)	0	0	2 (5.4%)
R	0	3 (15%)	0	3 (8.1%)
ISR + AVT	0	3 (15%)	0	3 (8.1%)
ISR + AVT+R	6 (66.7%)	14 (70%)	7 (87.5%)	27
Untreat	1 (11.1%)	0	0	1 (2.7%)
EBER-ISH (+)	8 (88.9%)	19 (95%)	7 (87.5%)	34 (91.9%)
Blood EBV DNA (+)	7 (77.8%)	16 (80%)	7 (87.5%)	30 (81.1%)
CMV infection (+)	1 (11.1%)	2 (10%)	2 (25%)	5 (13.5%)
GVHD	2 (22.2%)	1 (5%)	1 (12.5%)	4 (10.8%)
Acute	1 (11.1%)	1 (5%)	1 (12.5%)	3 (8.1%)
Chronic	1 (11.1%)	0	0	1 (2.7%)
Survival status				
Dead	1 (11.1%)	7 (35%)	2 (25%)	10 (27.0%)

*HP,* hyperplasias; *P-PTLD,* polymorphic PTLD; *DLBCL,* diffuse large B-cell lymphoma; *ISR,* immunosuppression reduction; *AVT,* antiviral therapy; *R,* rituximab; *EBER,* Epstein-Barr virus-encoded RNA; *EBV,* Epstein-Barr virus; *CMV,* cytomegalovirus; *GVHD,* graft-versus-host disease

## Discussion

PTLD was first described in patients after kidney transplantation in the 1960s [6], but the term “posttransplant lymphoproliferative disorder” or “posttransplant lymphoproliferative disease” (PTLD) was first introduced by Starzl in 1984 [7]. In 2018, Natkunam Y et al. proposed a new standardized nomenclature to address inconsistencies in terminology and diagnostic criteria for similar lesions across different immunodeficiency settings, aiming to guide iterative updates in the classification, disease definitions, and terminology of all immunodeficiency-associated lymphoproliferative disorders (IA-LPDs) [8]. The WHO-HAEM5 adopted this classification in 2022 [4]. PTLD is still among the serious complications of SOT and HSCT. The incidence of PTLD after bone marrow transplantation in recipients of HLA-matched related donors is approximately 1.0% and is as high as 25% in high-risk patients [9]. The incidence of PTLD in our cohort was 2.4% (37/1,544), which was higher than the 0.43% (78/18,014) reported in a large multicenter study [9] and may be attributable to the predominance of mismatched related donors in our transplant population.

Most HSCT recipients experience disease onset within 3 months after transplantation, with a median of 2 months after HSCT [10]. Our data revealed that 81.1% of the patients were diagnosed within 6 months after transplantation, and the most common disease duration ranged from 2 to 4 months (mean 3 months); the median time interval from transplantation to PTLD was 3 months. Notably, our cohort exhibited a complete absence of late-onset PTLD (conventionally defined as occurring later than 2 years post-transplantation), with all the cases emerging within 21 months [11]. This observation aligns with the well-established bimodal distribution of PTLD timing, which differs significantly between SOT and HSCT recipients [11]. In patients with SOT, late-onset PTLD is common and is often EBV negative. In contrast, PTLD following HSCT is characteristically an early complication, primarily because the period of most profound immunosuppression and, consequently, the highest risk for EBV-driven lymphoproliferation occurs in the first few months before immune reconstitution is achieved [12]. We acknowledge that our specific patient population and the study’s follow-up duration might have influenced this pattern. However, the overwhelming clustering of cases in the early posttransplant period is consistently reported in the HSCT literature; therefore, we believe that it accurately reflects the typical natural history of PTLD in this specific setting rather than being merely an artifact of our cohort selection or follow-up time.

The clinical manifestations of PTLD vary and lack specificity, but peripheral lymphadenopathy is often the first symptom. Most patients have fever and weakness before lymphadenopathy. The frequent sites of PTLD following HSCT are the lymph nodes, nasopharynx, tonsils and gastrointestinal (GI) tract [13]. Our data revealed that the sites were the lymph nodes, nasopharynx, tonsils and lungs. Once a patient has these nonspecific manifestations after transplantation, the possibility of PTLD should be considered, and shortening the time to diagnosis is very important.

The etiology of PTLD is complex. EBV infection is the most important risk factor and may be involved in the pathogenesis of PTLD [14, 15]. Nearly all PTLD cases following HSCT are EBV positive 16, 17]. Pathologically, ISH for EBV detection in biopsy tissue specimens is the recommended diagnostic method because of its high sensitivity and specificity [18]. In our cohort, the EBV positivity rate detected by EBER-ISH was 91.9% (34/37), which is consistent with the 91.4% reported in the literature [13]. Three patients were EBER negative, among whom two were also EBV DNA negative and one was EBV DNA positive (this patient died 2 months after disease onset). However, in some cases from our cohort, EBV-ISH demonstrated only sporadic positivity in individual cells, with two additional cases being negative. In such instances, correlation with clinical EBV DNA viral load testing is warranted. Although these laboratory data may be inconsistent, they still provide a valuable reference for pathological diagnosis. The diagnosis of PTLD should be based on biopsies of enlarged lymph nodes and other suspected sites [19]. However, early treatment is very critical for patients with PTLD. Waiting for pathological diagnosis may lead to a missed opportunity to cure PTLD. Because of rapid PTLD progression, clinicians opt for therapy at the same time or immediately after biopsy on the basis of a high EBV viral load and enlarged lymph nodes [20–22].

The WHO pathological classification of a tissue biopsy remains the gold standard for PTLD diagnosis [23]. On the basis of histological diagnosis, PTLDs can be classified into four major categories as follows: nondestructive PTLD, polymorphic PTLD, monomorphic PTLD, and classical Hodgkin lymphoma-type PTLD. However, the WHO-HAEM5 has introduced a new standardized nomenclature to address inconsistencies in terminology and diagnostic criteria for similar lesions in different immunodeficiency settings [4]. The lesions are referred to as lymphoid proliferations and lymphomas associated with immunodeficiency and dysregulation, including the following:

- (1) hyperplasias arising from immunodeficiency/dysregulation, with subtypes encompassing follicular hyperplasia (FH), IMH, and PCH;
- (2) polymorphic lymphoproliferative disorders arising from immunodeficiency/dysregulation and encompassing polymorphic posttransplant lymphoproliferative disorders;
- (3) EBV-positive mucocutaneous ulcers;
- (4) lymphomas arising from immunodeficiency/dysregulation, with subtypes encompassing diffuse large B-cell lymphoma, Burkitt lymphoma, classic Hodgkin lymphoma and T-cell lymphomas; and
- (5) inborn errors of immune-associated lymphoid proliferation and lymphoma.

Our research focused on lymphoproliferative disorders in a single immunocompromised state following HSCT. In accordance with the above histopathological classification, the cases included IMH, PCH, P-PTLD, and DLBCL. Notably, no cases of Burkitt lymphoma, classical Hodgkin lymphoma, plasmacytoma or FH were observed. The most common type was P-PTLD. The most common type was P-PTLD (54.0%), which is in line with the literature showing that P-PTLD is also the most frequent subtype (69.1%) [13]. This group represented a major category of diagnostically challenging cases in our patient series. P-PTLD includes heterogeneous lymphoid infiltrates containing variable proportions of B cells at all stages of differentiation, which results in architectural effacement of affected tissues. The infiltrates may include small lymphocytes, plasma cells, plasmacytoid cells and immunoblasts, as well as T cells and histiocytes. These lesions typically demonstrate monoclonal or oligoclonal populations, as evidenced by clonal IGH and/or IGK gene rearrangements. In our case series, there was a distinct category of lesions, which were predominantly composed of small lymphocytes, plasma cells, and plasmacytoid cells. In particular, we identified a subset of lesions that were characterized by a predominant population of small- to medium-sized cells. Immunohistochemically, these cells were positive for CD20 and plasma cell/plasmacytoid markers (CD79a, CD138, CD38 and MUM-1). Given these overlapping features, these lesions are often misdiagnosed as marginal zone lymphoma or plasmacytoma. In such challenging cases, ancillary immunohistochemical analysis is critical for achieving an accurate diagnosis. In some cases, P-PTLD requires differential diagnosis from DLBCL. The former lacks sheets of large atypical B cells. However, clonal immunoglobulin gene rearrangements may occasionally be detected in these patients.

While immunoglobulin gene rearrangements are characteristically associated with monomorphic PTLD (M-PTLD), we detected these genetic alterations in P-PTLD as well (Fig. 6), which is consistent with the data in the literature [24]. The underlying mechanisms may be associated with early EBV infection and/or an immunodeficient state [5]. Another finding in our cohort was the absence of detectable clonal T-cell populations, even though the literature suggests that approximately 50% of patients with B-cell PTLD harbor a concomitant monoclonal T-cell population [24]. We attribute these findings primarily to incomplete testing, as only 5 of our 37 patients underwent comprehensive TCRG and TCRB gene rearrangement analysis. Furthermore, in the two cases in which suspected TCR positivity was noted, the results were inconclusive, highlighting technical challenges in interpreting the results of clonality assays in patients with P-PTLD with a rich T-cell background. The preferential use of limited tissue material for B-cell clonality studies, which were deemed diagnostically more critical, likely further contributed to these findings. Therefore, the low rate of T-cell clonality detection in our study should not be interpreted as a true biological absence but rather as a limitation of the retrospective study design and clinical diagnostic priorities. In the three monomorphic DLBCL cases assessed by FISH, rearrangements of MYC, BCL2, and BCL6 were not detected, and no evidence of double-hit lymphoma (DHL) was detected. However, it should be noted that FISH analysis was performed selectively on DLBCL cases with medium-sized or blastoid morphology rather than on all DLBCL cases. Therefore, the negative results in these three selected cases do not exclude the possibility of such alterations in DLBCL patients lacking these morphological features, and our findings should not be interpreted as indicating the absence of double-hit/triple-hit lymphomas in the overall DLBCL cohort. Additionally, seven patients in our cohort with preexisting primary T-cell lymphoma who subsequently underwent transplantation developed P-PTLD (3 patients), PCH (3 patients) and DLBCL (1 patient). Given that these were of B-cell origin, the diagnosis was relatively uncomplicated. In contrast, distinguishing between T-cell PTLD and recurrent T-cell lymphoma posttransplantation poses significant diagnostic challenges. TCR gene rearrangement studies serve as essential ancillary tools for definitive classification. Since T-cell PTLD is very rare, further research is needed to accumulate more cases in this area. However, we recommend TCR clonality assessment as a critical diagnostic discriminator—the detection of TCR rearrangements that are identical to those in the original lymphoma indicates disease recurrence.

The therapeutic strategies for PTLD include reducing immunosuppression, surgical extirpation of localized disease, local radiation therapy, rituximab monotherapy, immunochemotherapy, chemotherapy, stem cell transplantation, and cellular immunotherapy [19, 25, 26]. Because of the rapid progression of PTLD, clinicians often choose therapy before definitive histological evidence is available [27]. Several preemptive approaches, including reducing immunosuppressive therapy, administering rituximab, and adoptively transferring EBV-specific T cells, have reduced the incidence of PTLD among both solid organ and hematopoietic stem cell transplant recipients [28, 29]. The benefits of rituximab are especially pronounced [30], but it can affect the morphology and immunophenotype of biopsy specimens.

Therefore, biopsy should be performed before initiating treatment. In our cohort, most patients received rituximab (administered weekly, 1–4 doses) after biopsy. The majority of patients showed significant clinical improvement. However, in one case, persistent lymphadenopathy was observed two months post-treatment, although a repeat biopsy revealed CD20 negativity. Furthermore, diminished or absent CD20 staining was noted in all five treated patients. These findings collectively underscore both the diagnostic necessity of pretherapeutic biopsy and the mechanistic efficacy of rituximab, as target depletion (evidenced by CD20 loss) is correlated with the treatment response.

Furthermore, the phenomenon of an altered postrituximab immunophenotype, as observed in one of our patients with the loss of both CD20 and EBER reactivity, warrants careful interpretation. While such changes are consistent with the intended therapeutic effect of depleting the target (CD20) and the associated viral reservoir (EBV), they result in a differential diagnosis. One must consider whether the results of a subsequent biopsy represent a recurrence of the original clone or the occurrence of a clonally distinct secondary malignancy. In our case, we favored the former interpretation, given the consistent histology of DLBCL and the persistence of other B-cell markers (PAX5 and CD79a) in both samples, which makes the diagnosis of coincidental, clonally unrelated DLBCL less likely.

Our study revealed a mortality rate of 27.0% (10/37), which is slightly higher than the 22.2% reported in the literature [13]; notably, no deaths were directly attributed to uncontrolled PTLD. Instead, mortality is driven primarily by infectious complications, which are well known consequences of the combined immunosuppressive state from underlying HSCT, GVHD, and PTLD-directed therapies, such as rituximab, and reduced immunosuppression. These findings underscore that the aggressiveness of PTLD in the HSCT setting may reflect not only the intrinsic biology of the tumor but also the patient’s vulnerability to treatment-related toxicity. The high infection-related mortality in our cohort, despite standard antimicrobial prophylaxis, further underscores the need for closer monitoring and more aggressive preventive strategies. These could include routine CMV surveillance, preemptive antifungal therapy, and immunoglobulin replacement, particularly in patients receiving rituximab and intensive immunosuppressive therapy. The rapid clinical decline in deceased patients (mean time to death of 3.5 months post-diagnosis) highlights the critical need for a multidisciplinary management strategy that aggressively balances PTLD control with proactive infection prevention and monitoring. Stratified analysis by histological subtype revealed that although the majority of patients received combined therapy with ISR, AVT, and rituximab, regardless of subtype, the outcomes differed considerably. Patients with hyperplasias had excellent outcomes (mortality: 11.1%), supporting the notion that these early lesional forms may be more responsive to immunotherapy and immunosuppression modulation alone. In contrast, the higher mortality in the P-PTLD (35.0%) and DLBCL (25.0%) groups suggests that more aggressive or prolonged therapy may be warranted in these subtypes. These findings highlight the clinical utility of accurate histological classification in guiding risk stratification and treatment intensity.

SEB specimens remain the gold standard for lymphoma diagnosis. However, in recent years, CNB has become widely adopted, posing diagnostic challenges for pathologists. In our cases, more than 60% of the lymph node specimens were obtained via CNB. While CNB performs well as a viable SEB alternative with high diagnostic accuracy, its utility remains limited for heterogeneous and polymorphic lesions [31]. While this approach is minimally invasive and technically straightforward, the limited tissue volume often complicates subsequent analyses, including IHC, ISH, and molecular testing. With respect to PTLD, SEB is similarly preferred, as it allows optimal evaluation of the nodal architecture. Although some studies suggest comparable diagnostic accuracy between CNB and excisional biopsy for lymph node evaluation, we strongly recommend SEB as the optimal approach for PTLD patients when feasible.

Several limitations of this study should be acknowledged. First, this was a single-center retrospective study with a relatively small sample size (*n* = 37), which limits the statistical power for subgroup analyses. Second, molecular workup, including FISH for MYC, BCL2, and BCL6 rearrangements and clonality analysis (both B-cell and T cell), was not performed in all cases, primarily because of limited tissue availability from CNB specimens. Third, the median follow-up time was 15 months, and the retrospective nature of the study may have led to underdetection of late-onset PTLD or late relapses. Despite these limitations, our study provides a detailed clinicopathological characterization of PTLD in our HSCT cohort and highlights key diagnostic challenges in routine practice.

## Conclusion

In summary, we report a single-center series of patients with PTLD after HSCT, representing a morphologically heterogeneous spectrum of EBV-driven lymphoid proliferations, which are characterized by disseminated disease, rapid progression, a fulminant clinical course, and high mortality. Histomorphology, supplemented by immunohistochemistry and EBER in situ hybridization, remains the cornerstone for diagnosing PTLD. Our study highlights the importance of pathologists developing a deep understanding of this disease and closely correlating pathological findings with clinical data. Furthermore, enhancing collaboration with the ultrasound puncture team is crucial for obtaining adequate biopsy material for comprehensive diagnostic evaluation, which also facilitates future research and enables more accurate pathological diagnosis.

## Data Availability

No datasets were generated or analysed during the current study.
